# Prevalence of CTX-M *β*-Lactamases Producing Multidrug Resistant *Escherichia coli* and *Klebsiella pneumoniae* among Patients Attending Bir Hospital, Nepal

**DOI:** 10.1155/2021/9958294

**Published:** 2021-06-08

**Authors:** Sushma Koirala, Sujan Khadka, Sanjeep Sapkota, Suprina Sharma, Santosh Khanal, Alina Thapa, Dhruba Kumar Khadka, Pramod Poudel

**Affiliations:** ^1^Department of Microbiology, National College (NIST), Tribhuvan University, Kathmandu, Nepal; ^2^State Key Laboratory of Environmental Aquatic Chemistry, Research Center for Eco-Environmental Sciences, Chinese Academy of Sciences, Beijing 100085, China; ^3^University of Chinese Academy of Sciences, Beijing 100049, China; ^4^State Key Laboratory of Respiratory Disease, Guangzhou Institutes of Biomedicine and Health, Chinese Academy of Sciences, Guangzhou 510530, China; ^5^State Key Laboratory of Alpine Ecology and Biodiversity, Institute of Tibetan Plateau Research, Chinese Academy of Sciences, Beijing 100101, China; ^6^Department of Microbiology, Bir Hospital, National Academy of Medical Sciences (NAMS), Kathmandu, Nepal; ^7^Central Department of Biotechnology, Tribhuvan University, Kirtipur 44618, Nepal; ^8^Research Division, University Grants Commission (UGC), P.O. Box: 10796, Sanothimi, Bhaktapur, Nepal

## Abstract

The emergence of multidrug resistant (MDR) bacteria which is attributable to extended spectrum *β*-lactamases (ESBLs) production of CTX-M types is an obvious problem worldwide. This study is aimed at determining the prevalence of CTX-M *β*-lactamases producing multidrug resistant *Escherichia coli* and *Klebsiella pneumoniae* among patients attending Bir Hospital. A cross-sectional study was conducted between April and September 2019 at Bir Hospital, Kathmandu, and Department of Microbiology, National College, Kathmandu, Nepal. A total of 5,690 different clinical specimens were subjected to cultural, microscopic, and biochemical analyses for the identification of the isolates. Antimicrobial susceptibility testing of the isolates was done, and MDR isolates were selected and processed for further ESBL confirmation by the combination disks method. All confirmed ESBL isolates were screened for CTX-M type *β*-lactamases (*bla*_CTX-M_) by PCR. Of the total 345 isolates (227 *Escherichia coli* and 118 *Klebsiella pneumoniae*), 232 were MDR. All 232 (67.24%) MDR isolates were suspected as ESBL producers on the screening test. However, on the phenotypic test, 135 (58.18%) of total MDR bacteria were confirmed as ESBL producers with the highest proportion in *K. pneumoniae* (59.37%). The major source of ESBL producers was urine. ESBL producing isolates were mostly identified from outpatients and patients belonging to age group 41-60. Gentamicin was found to be effective against ESBL producers. The prevalence of *bla*_CTX-M_ was (89.62%) with the highest frequency for *E. coli* (93.81%). High prevalence of ESBL of CTX-M types among MDR *E. coli* and *K. pneumoniae* was detected from clinical specimens of patients in Bir Hospital. This study warrants the need for the judicious use of antibiotics as well as emphasize the use of modern diagnostic tools for the early detection of MDR and ESBL producers to curb the emergence and spread of MDR and ESBL producing bacteria in the clinical settings of Nepal.

## 1. Introduction

The enormous increases in multidrug resistant (MDR) strains have become a worldwide challenge and create therapeutic difficulties in selecting proper antimicrobial drugs [1]. MDR bacteria are those that have the prowess to cause resistance to at least one agent of three different classes of common antimicrobial agents [2]. During the very important discovery of antibiotics, based on the hypothesis that the persistence of the resistance to antibiotics due to mutation was negligible, it was assumed that the progression of antimicrobial resistance was avoidable [3]. Nowadays, however, various mechanisms have been documented that are ascribable to develop resistance to several antimicrobials agents by organisms [4].

The production of ESBLs is recognized as one of the several mechanisms to expand resistance in Enterobacteriaceae [5]. ESBLs are the *β*-lactamases enzymes that can cause resistance to *β*-lactam antibiotics (by damaging *β*-lactam rings) and other monobactam antibiotics such as aztreonam but are sensitive to *β*-lactamase inhibitors and cephamycins [6]. Although ESBL was discovered several decades ago, there have been troublesome to sort out organisms that are responsible for ESBL production due to several reasons such as difficulty in its detection and variability during reporting [7]. A large number of studies have reported the presence of ESBL producers in clinical [8–10] as well as nonclinical samples [11–13]. Nevertheless, some studies concluded *E. coli* and *K. pneumoniae* as the most crucial bacteria responsible for ESBL production [14, 15].

MDR and *β*-lactamases producing *E. coli* and *K. pneumoniae* have been undoubtedly the most frequently studied topics every year. An explosive increase of *β*-lactamases has been described globally, and this increase is due to class A and D *β*-lactamases [16]. Class A *β*-lactamases are classified into three common types: TEM, SHV, and CTX-M [17]. More than 193 SHV types, 223 TEM types, and 172 CTX-M types are identified till now [1]. CTX-M type ESBL belongs to Ambler's class A/Bush's group 2be and comprises nonhomogeneous and complex groups of enzymes [5]. According to Bush and Jacoby [16], the TEM, SHV, and OXA type ESBL enzymes were derived from the alteration in single base pair whereas CTX-M type ESBL enzymes derived through the transition of chromosomal *β*-lactamases genes from *Kluyvera* species when they were incorporated into mobile genetic elements [5].

Based on the sequencing of amino acid, CTX-M has been classified into five lineages across pathogens: CTX-M-1, CTX-M-2, CTX-M-8, CTX-M-9, and CTX-M-25 [1]. CTX-M type ESBL, which have been predominant since 2005 [18], was first identified in Germany in 1989 [19]. After its predominant, it started leading to rising of carbapenem resistant Enterobacteriaceae due to excessive use of carbapenem for its treatment [20]. Nowadays, CTX-M type ESBLs which are mostly plasmid integrated but chromosome integrated occasionally are reported as predominant than SHV and TEM in both developed and developing nations [21]. CTX-M-15 is the most commonly found CTX-M in human pathogens across the globe followed by CTX-M-14 [21].

Several studies in Nepal have reported the prevalence of MDR [22, 23] and ESBL producers [8–10]. Despite knowing the fact, in Nepal, the literature regarding ESBL production among MDR isolates and their responsible genes are poorly stated than other developed nations; the present study would help to investigate the prevalence of CTX-M *β*-lactamases producing MDR *E. coli* and *K. pneumoniae* among patients.

## 2. Materials and Methods

### 2.1. Study Period, Design, and Setting

This was a hospital-based cross-sectional study conducted between April and September 2019 at Bir Hospital, Kathmandu, and the Department of Microbiology at National College, Kathmandu. Bir Hospital is a tertiary hospital, located in the heart of Kathmandu City with accurate geographic coordinates (27.705053°N, 85.313608°E). The hospital has 458 beds and provides care for more than 45,000 patients per year.

### 2.2. Sample Size, Processing, and Identification

A total of 5,690 different clinical specimens that included urine (*n* = 2,710), sputum (*n* = 1,490), pus (*n* = 770), blood (*n* = 512), and body fluids (*n* = 208) were cultured on different agar media such as nutrient agar, MacConkey agar, cysteine–lactose electrolyte deficient medium, 5% sheep blood agar, chocolate agar, and brain heart infusion broth as well as bile broth (HiMedia, Mumbai, India) depending upon requirements and isolated following standard microbiological techniques [24]. The identification of *E. coli* and *K. pneumonia* was done by using standard microbiological techniques, which involved studying the colonial morphology, Gram staining, and various biochemical tests (indole, methyl-red, Voges-Proskauer, citrate utilisation, triple sugar iron, oxidase, catalase, oxidative/fermentative, motility, and urease) [24].

### 2.3. Antimicrobial Susceptibility Testing

Antibiotic susceptibility test (AST) of both clinical isolates was performed using the modified Kirby-Bauer disk diffusion method on Mueller Hinton agar (Hi-Media Laboratories, India) following standard zone size interpretative criteria set by the Clinical and Laboratory Standards Institute (CLSI) [25]. The different antibiotic disks used in this study during AST were procured from HiMedia Laboratories, India, and include amoxicillin (30 *μ*g), gentamicin (10 *μ*g), cotrimoxazole (25 *μ*g), ciprofloxacin (5 *μ*g), imipenem (10 *μ*g), amoxicillin/clavulanic acid (20/10 *μ*g), cefotaxime (30 *μ*g), ceftriaxone (30 *μ*g), ceftazidime (30 *μ*g), aztreonam (30 *μ*g), and cefpodoxime (10 *μ*g). The *E. coli* and *K. pneumoniae* isolates were regarded as MDR isolates if they were resistant to at least one agent of three different classes of antimicrobial disks [2].

### 2.4. Phenotypic Detection of ESBL Producers

For the screening of production of ESBL, cefotaxime (30 *μ*g), ceftazidime (30 *μ*g), ceftriaxone (30 *μ*g), aztreonam (30 *μ*g), and cefpodoxime (10 *μ*g) were used [25]. The MDR isolates are suspected to be the ESBL producers if they are resistant to one or all of the aforementioned drugs [25]. The suspected isolates were confirmed by combination disks test using ceftazidime (30 *μ*g) versus ceftazidime/clavulanic acid (30/10 *μ*g) discs and cefotaxime (30 *μ*g) versus cefotaxime/clavulanic acid discs (30/10 *μ*g). ESBL production was confirmed if the zone of diameter was ≥5 mm in the clavulanic acid disk when compared to the individual disk.

### 2.5. Genomic DNA Extraction and Polymerase Chain Reaction (PCR) Amplification

The bacterial DNA was extracted using the phenol-chloroform assay [26]. PCR was performed targeting the *bla*_CTX-M_ gene of *E. coli* and *K. pneumoniae* isolates. PCR reaction mixture was prepared by mixing 6 *μ*L nuclease-free water, 2 *μ*L master mix, 0.5 *μ*L forward primer, 0.5 *μ*L reverse primer, and 1 *μ*L DNA template in a sterile PCR tube. The *bla*_CTX-M_ was amplified with the set of primer: CTX-M-F-5′–TCTTCCAGAATAAGGAATCCC–3′ and CTX-M-R-5′–CCGTTTCCGCTATTACAAAC–3′ with 909 bp amplicon size [27]. PCR amplification was done on a thermocycler (TaKaRa, Tokyo, Japan) with conditions: initial denaturation at 94°C for 5 min, denaturation at 94°C for 30 s, annealing at 55°C for 30 s, extension at 72°C for 1 min, followed by terminal extension at 72°C for 5 min; the reaction was 29 cycles. The gel was prepared by mixing 1% agarose gel powder in 1X TAE (tris-acetate-EDTA) solution. Then, 0.5 *μ*L EtBr (ethidium bromide) was added to the mixture and mixed well and was poured into the electrophoresis tank. The comb was set appropriately and allowed to solidify. After gel preparation, 6 *μ*L of 100 bps ladder and 4 *μ*L of PCR product were added to the flanking lane of the well. About 70 V of power was supplied for 45 min. Finally, the gel was taken for photo documentation in a UV transilluminator (BioRad, USA).

### 2.6. Quality Control

In this study, the standard aseptic procedures were employed, and all the batches of cultural media and chemical reagents were processed following the CLSI protocols. The control strains of *E. coli* (ATCC 25922) and *K. pneumoniae* (ATCC 700603) were used to adjust the quality control of AST. Using both *K. pneumoniae* and *E. coli* harboring *bla*_CTX-M_ gene during PCR, the positive control was maintained, whereas the negative control for both isolates was assured by using nuclease-free water.

### 2.7. Data Management and Statistical Analysis

All the data obtained were analysed using the R-programming statistical analysis tool (version 1.2.5033) and the Statistical Package for Social Sciences (SPSS) software (version 16.0). Chi-square (*χ*2) test was estimated, and *P* < 0.05 was considered statistically significant.

## 3. Results

### 3.1. Growth Pattern of Isolates

**Figure 1:**
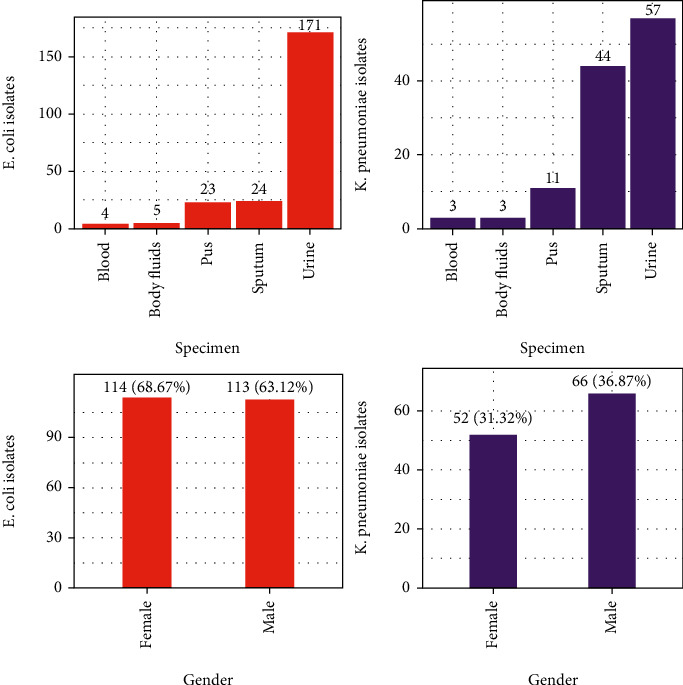
Distribution of isolates with respect to specimens and gender (*n* = 345). Note: body fluids include cerebrospinal fluid, peritoneal fluid, pleural fluid, and synovial fluid.

Out of 5,690 clinical specimens processed, growth was detected in 20.07% (1,142) specimens. Among total growth, Gram-negative accounts for 879 (76.97%) isolates of which 227/879 (25.82%) were *E. coli* and 118/879 (13.42%) *K. pneumoniae* indicating *E. coli* as a predominant bacterium. The greater number of isolates 179/345 (51.88%) was recovered from male patients; however, *E. coli* was identified more from females (68.67%). Similarly, the highest percentage of the isolates was from urine samples 228/345 (66.08%). Moreover, the highest percentage of *E. coli* (75%) and *K. pneumoniae* (48.30%) was isolated from urine (Figure 1).

### 3.2. Antibiotic Susceptibility Pattern of Bacterial Isolates

**Figure 2:**
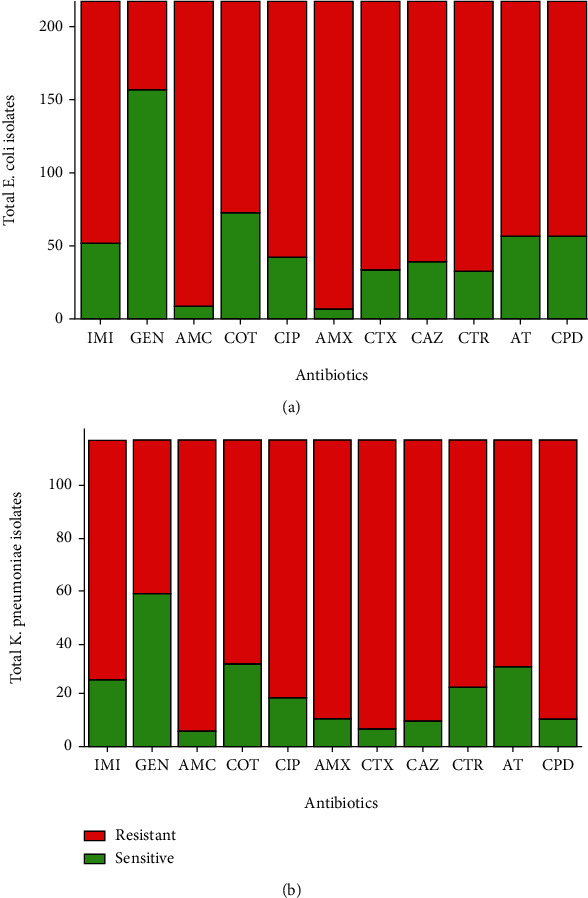
(a, b) Antibiotic susceptibility patterns of *E. coli* and *K. pneumoniae*, respectively. IMI: imipenem; GEN: gentamicin; AMC: amoxicillin/clavulanic acid; COT: cotrimoxazole; CIP: ciprofloxacin; AMX: amoxicillin; CTX: cefotaxime; CAZ: ceftazidime; CTR: ceftriaxone; AT: aztreonam; CPD: cefpodoxime.

**Figure 3:**
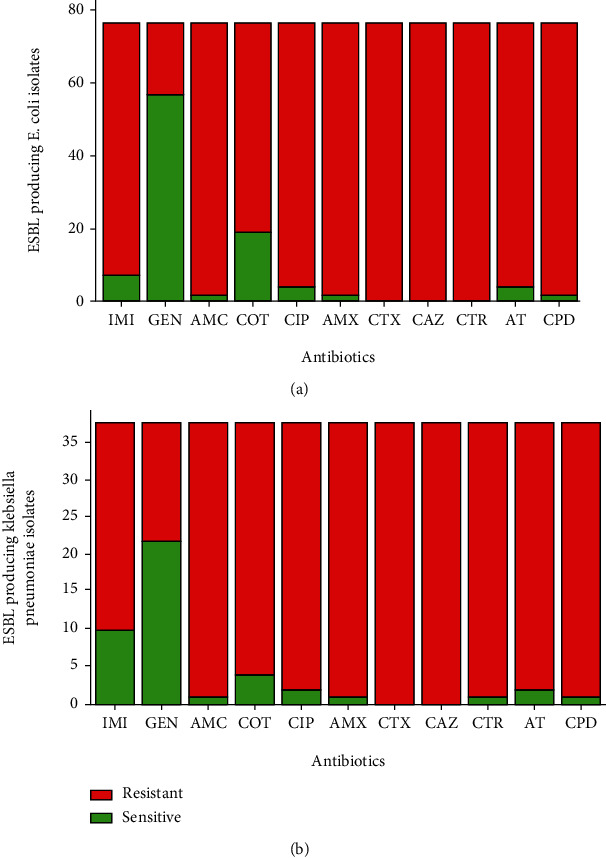
(a, b) Antibiotic susceptibility patterns of ESBL producing *E. coli* and *K. pneumoniae*, respectively. IMI: imipenem; GEN: gentamicin; AMC: amoxicillin/clavulanic acid; COT: cotrimoxazole; CIP: ciprofloxacin; AMX: amoxicillin; CTX: cefotaxime; CAZ: ceftazidime; CTR: ceftriaxone; AT: aztreonam; CPD: cefpodoxime.

*In vitro* drug susceptibility was performed for all the isolates by the modified Kirby-Bauer disc diffusion method. The highest sensitivity for total isolates was found towards gentamicin which was only 164/227 (72.24%) and 59/118 (50%) for *E. coli* and *K. pneumoniae*, respectively, whereas the least susceptibility was towards amoxicillin followed by amoxicillin/clavulanic acid for both isolates (Figures 2(a) and 2(b)). Similarly, the highest rate of sensitivity for ESBL producing *E. coli* and *K. pneumoniae* was towards gentamicin comprising 72/97 (74.22%) and 22/38 (57.89%), respectively. All ESBL producing *E. coli* isolates were found to be (100%) resistant towards cefotaxime, ceftazidime, and ceftriaxone. A similar rate of drug resistivity as in ESBL producing *E. coli* was also observed in ESBL producing *K. pneumoniae* isolates where they were found to be 100% resistant towards cefotaxime and ceftazidime. Those isolates which showed resistance to at least one agent of three different classes of antibiotics were regarded as MDR isolates (Figures 3(a) and 3(b)).

### 3.3. Specimen Wise Distribution of Multidrug Resistant Strains

**Figure 4:**
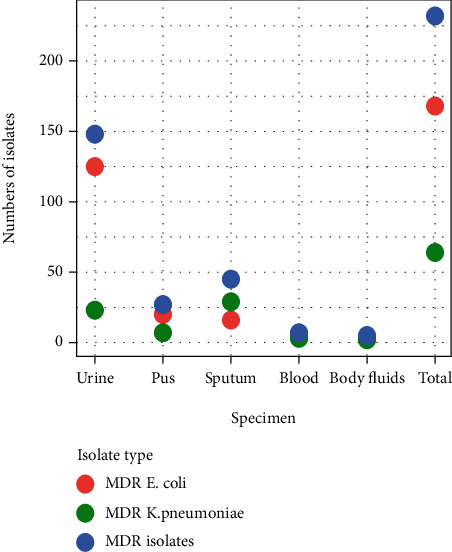
Specimen wise distribution of multidrug resistant isolates.

Multidrug resistance was observed in 232/345 (67.24%) isolates. Overall, 168/345 (48.69%) of *E. coli* and 64/345 (18.55%) of *K. pneumoniae* were MDR. Of the 232 MDR cases, the highest multidrug resistance pattern (72.41%) was detected in *E. coli* compared to *K. pneumoniae* (27.58%). Although the least number of isolates was isolated from blood, it accounts for 100% of MDR isolates. The highest proportion of multidrug resistance in *E. coli* was reported from pus 20/227 (58.82%). On the other hand, the highest proportion of multidrug resistance in *K. pneumoniae* was reported from blood 3/7 (42.85%) (Figure 4).

### 3.4. Prevalence of ESBL and *bla*_CTX-M_ among Multidrug Resistant Isolates

**Figure 5:**
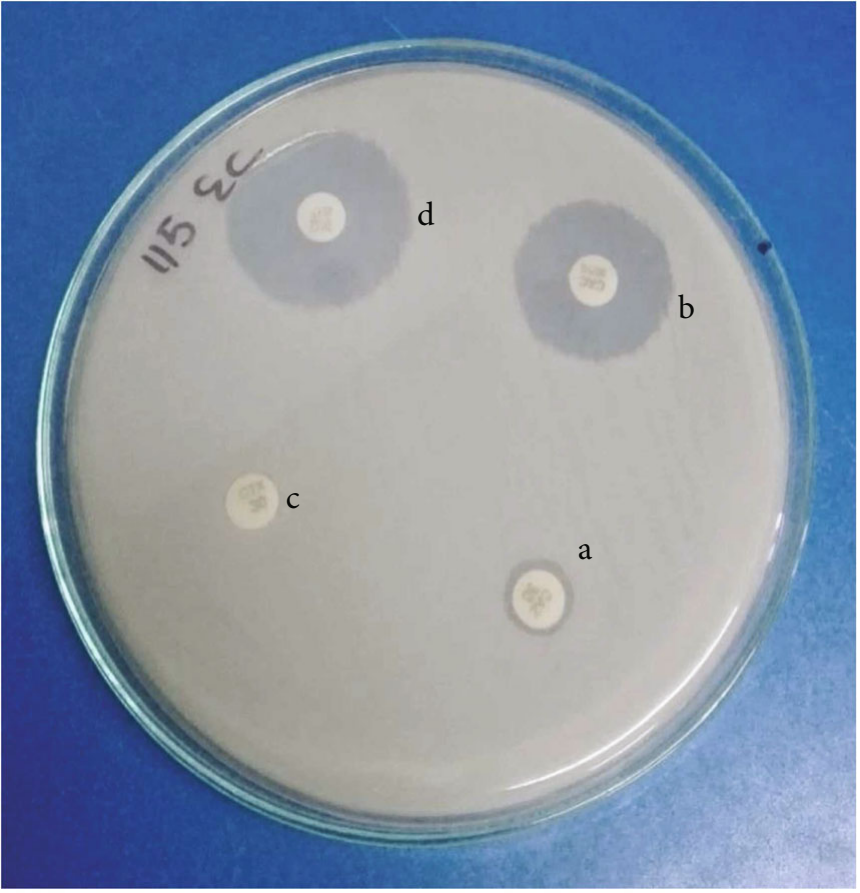
Phenotypic confirmation of ESBL isolates by combined disk method: (a) ceftazidime; (b) ceftazidime/clavulanic acid; (c) cefotaxime; (d) cefotaxime/clavulanic acid.

**Figure 6:**
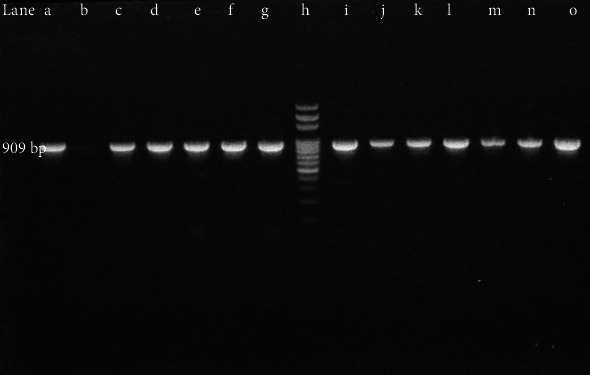
Amplification of *bla*_CTX-M_ gene (*E. coli*). Lane (a) positive control; lane (b) negative control (nuclease-free water); lanes (c–g) *bla*_CTX-M_ positive *E. coli*; lane (h) 100 bp ladder; lanes (i–o) *bla*_CTX-M_ positive *E. coli* isolates.

**Figure 7:**
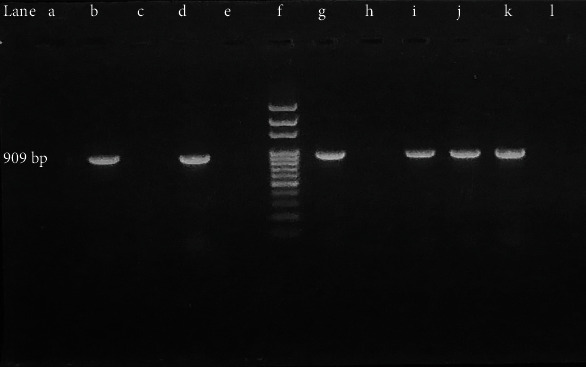
Amplification of *bla*_CTX-M_ gene (*K. pneumoniae*). Lane (b) positive control; lane (c) negative control (nuclease-free water); (a, e, h, l) *bla*_CTX-M_ negative *K. pneumoniae*; lane (f) 100 bp ladder; lanes (d, g, i, j, k) *bla*_CTX-M_ positive *K. pneumoniae* isolates.

ESBL prevalence among total isolates was 135/345 (39.13%) whereas ESBL prevalence among MDR isolates was 135/232 (58.18%) even though 100% of isolates exhibited screening positive consequences. The phenotypic confirmation of ESBL producing isolates is depicted in Figure 5. Despite having a large number of ESBL isolates (97/135) among total ESBL in *E. coli* isolates, the percentage of ESBL isolates among individual MDR strains was highest in *K. pneumoniae* 38/64 (59.37%) indicating *K. pneumoniae* as the most frequent ESBL producer than *E. coli*. When 135 ESBL isolates were tested for detection of *bla*_CTX-M_ gene using PCR, the overall prevalence of *bla*_CTX-M_ gene was 121 (89.62%). The percentage of *bla*_CTX-M_ gene among each ESBL bacteria was 91/97 (93.81%) among *E. coli* and 30/38 (78.94%) among *K. pneumonia* (Table 1). PCR amplification of *bla*_CTX-M_ gene is shown in Figures 6 and 7. The *bla*_CTX-M_ was confirmed from ESBL isolates of different clinical specimens comprising 82/121 (67.76%) from urine, 14/121 (11.57%) from pus, 15/121 (12.39%) from sputum, 7/121 (5.78%) from blood, and 3/121 (2.47%) from body fluids.

### 3.5. Distribution of ESBL Isolates with respect to Different Variables

Despite having a greater number of ESBL isolates 69/135 (51.11%) among male patients, ESBL isolates of *E. coli* were identified more from females (*P* > 0.05). A large number of ESBL isolates of *K. pneumoniae* were identified from inpatients even though the majority of total ESBL isolates 116/135 (85.92) were isolated from outpatients (*P* > 0.05). More number of ESBL isolates were isolated from urine comprising 93/135 (68.88%) isolates. The largest percentage of ESBL producing *E. coli* was isolated from body fluids, and *K. pneumoniae* was isolated from sputum (*P* < 0.05). Overall, the patients with an age range from 41 to 60 were affected mostly by these isolates 45/135 (33.62%). On the contrary, ESBL producing *E. coli* was more common among patients with age 21-40 (*P* > 0.05) (Table 2).

## 4. Discussion

Out of total 5,690 clinical specimens processed, growth was detected in 20.07% of specimens. This interpretation is comparable to the interpretation by Nepal et al. [28] and Ghimire et al. [6] where growth was detected in 17.10% and 17% specimens. Among total growth, Gram-negative accounts for 879 (76.97%) isolates of which *E. coli* was 25.82% whilst *K. pneumoniae* was 13.42% of total Gram-negative isolates. The predominance of *E. coli* in this study accords with the predominance of *E. coli* in the other studies [28, 29]. The majority of isolates (51.88%) were isolated from the male which is unparallel to the result drawn by Parajuli et al. [30]. The plausible reason may be that the males have routine outdoor work and are more likely to get the infection from the infected environments. The highest magnitude of isolates was isolated from urine (66.08%) which is consistent with the study done in Nepal [28]. The reason for the highest prevalence of isolates from urine is due to the inclusion of larger numbers of urine samples in this study.

In the current study, both *E. coli* and *K. pneumoniae* shared several similarities in the AST profile. There was a limited number of drug sensitivity for both ESBL producer as well as non-ESBL producer, and the drug of choice was gentamicin. This finding was not in harmony with the result of other studies [28, 31] where imipenem had higher sensitivity. The percentage of sensitivity towards gentamicin in this study, however, is very low as compared to the study performed in Nepal [15] where the sensitivity of gentamicin was 89.40% for *E. coli* and 100% for *K. pneumoniae*. The low susceptibility to gentamicin is due to posttranscriptional modification and mutation of bacteria [32]. It was found that all isolates of ESBL producing *E. coli* were 100% resistant to the third-generation cephalosporin, cefotaxime, ceftazidime, and ceftriaxone which is in tune with the finding of Pokhrel et al. [33] and Dahal et al. [34]. Likewise, the 100% resistance of ESBL producing *K. pneumoniae* to cefotaxime and ceftazidime is in line with the finding of Nepal et al. [28]. However, screening test may not be reliable if ceftazidime is solely used as a screening agent because CTX-M producing isolates have a specific tendency towards cefotaxime and can be sensitive towards ceftazidime during the test [35].

Amoxicillin which also has a higher resistance rate (>90%) towards both ESBL and non-ESBL isolates of *K. pneumoniae* and *E. coli* in our investigation showed similar finding with Shakya et al. [15]. Similarly, >90% resistance to aztreonam and cefpodoxime was observed which specify them to be effective for ESBL screening but not as much as cefotaxime and ceftazidime. Moreover, >75% resistance to cotrimoxazole and amoxicillin/clavulanic acid for both ESBL isolates in this study is in tune with the finding of Shashwati et al. [31] indicating them to be unsuitable for these infections. Both *E. coli* and *K. pneumoniae* showed more than 80% resistance to ciprofloxacin which might be due to mutation at the target site, i.e., *gyrA* and *parC* [36].

One of the noteworthy findings in the present study among ESBL producers was the high resistance rate of 90.72% for *E. coli* and 73.68% for *K. pneumoniae* to last-resort antibiotics (imipenem) which is contrary to that found in Shakya et al. [15] and Zeynudin et al. [37] who reported that the imipenem as 0% and 1.90% resistant, respectively. Parajuli et al. [30] also described 100% and 93% sensitivity of *E. coli* and *Klebsiella* species towards imipenem which is unparallel to our finding. This uncommon resistance to imipenem in our finding is attributable to the increase in the haphazard use of the last-resort drug to treat severe infections and the presence of carbapenemase *β*-lactamases [6].

Due to the difference in antibiotics prescribing habits during infection and lapse in an effective program for infection control, multidrug resistance patterns may vary from country to country or among the hospitals even in the same country. Multidrug resistance was observed in 67.24% of isolates which is less compared to the study carried by Ghimire et al. [6]. The reason for maximum multidrug resistance is due to mutation in chromosomal genes [6]. The prevalence of MDR isolates was higher among *E. coli* (74%) than *K. pneumoniae* (54.23%) which disaccords to the study by Ghimire et al. [6] where the prevalence of MDR isolates was reported 75% and 94.40%, respectively. Blood specimens account for a 100% prevalence of MDR bacteria. For the screening of production of ESBL, cefotaxime, ceftazidime, ceftriaxone, aztreonam, and cefpodoxime disks were used. Hundred percentage MDR isolates were suspected as possible ESBL producers which is unparallel to the finding of Teklu et al. [38] which reported only 62.20% MDR isolates as ESBL producers. This could be explicated by the difference in the exploitation of cephalosporins in prophylaxis amidst the respective setups [39].

The prevalence of ESBL ranges from <1% to >70% throughout the world [15]. This difference may be due to different geographic locations, differences in the program conducted for appropriate use of antibiotics, and control measures [30]. The overall ESBL prevalence of 39.13% among total isolates in the study is similar to that of other comparable studies [6, 30] where ESBL prevalence was 40.60% and 38.80%, respectively. However, this rate of ESBL prevalence is higher than the study by Raut et al. [40] where ESBL prevalence was 22.4% and lower than the study by Abrar et al. [29] where ESBL prevalence was 79%. ESBL prevalence of 58.18% among MDR isolates can be comparable to the result obtained by Ghimire et al. [6] where 47% of ESBL isolates were prevalent among MDR isolates which is slightly lower than our reporting. In this study, a high prevalence of ESBLs might be due to biases of specimens. The largest percentage of ESBL production among *K. pneumoniae* (59.37%) than *E. coli* (57.73%) agreed with the finding of Teklu et al. [38].

Regarding gender, a greater number of ESBL producers (51.11%) were isolated from the male which shows similarity to the interpretation in other studies [29, 31]. The slight female predominance with 50.51% isolates was noticed in *E. coli* which is less when compared to 58.5% *E. coli* among females by Teklu et al. [38]. The appropriate reason for female predominance is that females are more vulnerable to community-acquired infections [29, 41]. The male predominance with 55.26% isolates was noticed in *K. pneumoniae* which signifies that males are more prone to hospital-acquired infection [29, 41]. A remarkable difference between inpatients and outpatients was found with ESBL isolates more presenting in outpatients (85.92%) corresponds to those reported by Nepal et al. [28] and was different to those reported by Parajuli et al. [30]. The presence of ESBL isolates more in outpatients in this study indicates the spreading of ESBL producers in the community setting. The urinary isolates were the most common ESBL producing isolates, i.e., 68.88% which correlates with the finding of Parajuli et al. [30] in which the urinary isolates were 51.60%. The reason for this is due to the larger number of urine specimens collected for microbiological analysis during the study. The high prevalence of the ESBL producers among patients with age group 41-60 was observed, and the reasons behind it remain unclear. The previous study by Ben-Ami et al. [41] reported high ESBL infection rates in old age individuals.

Since the past decade, CTX-M positive *E. coli* and *K. pneumoniae* are the most prevalent ESBL producers throughout the world and particularly in several Asian countries [15]. A higher proportion of *bla*_CTX-M_ was present among *E. coli* (93.81%) than *K. pneumoniae* (78.94%) which seemed to have contrary to the report of Parajuli et al. [30] where *bla*_CTX-M_ was present on 100% *K. pneumoniae* and 90.60% *E. coli*. Since, *bla*_CTX-M_ is the most predominant genotype among ESBL genotype [14]; the overall prevalence of the *bla*_CTX-M_ gene was 89.62% which concordance with the report of Parajuli et al. [30] where the *bla*_CTX-M_ gene was prevalent on 91.40% of isolates. But the prevalence was found higher when compared to the study done by Abrar et al. [29] where *bla*_CTX-M_ was prevalent on 76% of isolates. The occurrence of CTX-M types ESBL differs based on geographic locations [21].

## 5. Conclusions

This study demonstrates the higher level of multidrug resistance as well as ESBL production among the clinical isolates. The most accustomed antibiotics for the ESBL producers were found to be ineffective against the highest proportion of ESBL isolates; however, gentamicin was effective against them. The prevalence of *bla*_CTX-M_ was high with a higher frequency for *E. coli*. ESBL producing isolates showed higher resistance to imipenem, a last-resort drug to cure an infection caused by ESBL producer. Thus, it ensures the dire need for rational use of antibiotics in the clinical settings of Nepal. The dramatic increase of ESBL among MDR bacteria can be minimized by making compulsory detection of ESBL producing pathogens in daily practice for every laboratory, by expanding the ESBL research for the identification of resistance mechanisms, by conducting AMR (antimicrobial resistance) control programs, and by determining effective measures for infection control.

## Figures and Tables

**Table 1:** Prevalence of ESBL and *bla*_CTX-M_ among multidrug resistant isolates.

Isolates	Total isolates	MDR isolates *n* (%)	ESBL screening positive among MDR isolates *n* (%)	ESBL positive among MDR isolates *n* (%)	*bla*_CTX-M_ among ESBL producers *n* (%)
*E. coli*	227	168 (74.01)	168 (100)	97 (57.73)	91 (93.81)
*K. pneumoniae*	118	64 (54.24)	64 (100)	38 (59.37)	30 (78.94)
Total	345	232 (67.25)	232 (100)	135 (58.18)	121 (89.62)

**Table 2:** Distribution of ESBL producing isolates with respect to different variables.

Variables	ESBL isolates *n* (%)	ESBL producing		*P* value
		*E. colin* (%)	*K. pneumoniaen* (%)	
Gender				
Male	69 (51.11)	48 (49.49)	21 (55.26)	0.545
Female	66 (48.88)	49 (50.51)	17 (44.74)	
Patients' type				
Inpatients	19 (14.07)	12 (12.37)	7 (18.42)	0.363
Outpatients	116 (85.92)	85 (87.63)	31 (81.58)	
Specimens' type				
Urine	93 (68.88)	74 (76.29)	19 (50.00)	0.000^∗^
Pus	15 (11.11)	12 (12.37)	3 (7.89)	
Sputum	17 (12.59)	4 (4.12)	13 (34.21)	
Blood	7 (5.78)	4 (4.12)	3 (7.89)	
Body fluids	3 (2.22)	3 (3.09)	0 (0.00)	
Age group				
≤20	8 (6.08)	5 (5.16)	3 (10.74)	0.925
21-40	32 (24.05)	24 (24.74)	8 (21.05)	
41-60	45 (33.62)	31 (31.95)	14 (36.84)	
61-80	39 (28.98)	29 (29.89)	10 (26.32)	
≥81	11 (7.24)	8 (8.24)	3 (10.74)	

^∗^ indicates statistically significant at 5% level of significance.

## Data Availability

Upon request, the corresponding author will provide the data used to support the results of this study.

## Abbreviations

AMR: Antimicrobial resistance
AST: Antimicrobial susceptibility test
MDR: Multidrug resistant
ESBL: Extended spectrum *β*-lactamase
*bla*_CTX-M_: *β*-Lactamase cefotaximase Munich
CLSI: Clinical and laboratory standard test.

## Additional Points

*Strengths and Limitations of the Study*. In the context of Nepal, very limited studies regarding the isolation of the *bla*_CTX-M_ gene from the *E. coli* and *K. pneumoniae* were conducted in the clinical as well as nonclinical settings using both phenotypic as well as molecular tests. This study could pave the way for the policymakers of this country to work on formulating effective antimicrobial policies, robust treatment and diagnostic procedures, and proper management of diseases in the clinical settings. However, this research possesses some limitations such as the study being confined to a single hospital, short duration of the research, and limited exploration of the *β*-lactamases. Thus, future studies should research all *β*-lactamases in multiple hospitals for a longer period to overcome the present drawbacks.

## References

[1] Ramadan A. A., Abdelaziz N. A., Amin M. A., Aziz R. K. (2019). Novel _bla_ CTX-M variants and genotype-phenotype correlations among clinical isolates of extended spectrum beta lactamase-producing _Escherichia coli_. *Scientific Reports*.

[2] Magiorakos A. P., Srinivasan A., Carey R. B. (2012). Multidrug-resistant, extensively drug-resistant and pandrug-resistant bacteria: an international expert proposal for interim standard definitions for acquired resistance. *Clinical Microbiology and Infection*.

[3] Davies J. (1994). Inactivation of antibiotics and the dissemination of resistance genes. *Science*.

[4] van Hoek A. H. A. M., Mevius D., Guerra B., Mullany P., Roberts A. P., Aarts H. J. M. (2011). Acquired antibiotic resistance genes: an overview. *Frontiers in Microbiology*.

[5] Cantón R., González-Alba J. M., Galán J. C. (2012). CTX-M enzymes: origin and diffusion. *Frontiers in Microbiology*.

[6] Ghimire A., Acharya B., Tuladhar R. (2018). Extended spectrum *β*-lactamase (ESBL) producing multidrug resistant Gram-negative bacteria from various clinical specimens of patients visiting a tertiary care hospital. *Tribhuvan Univ. J. Microbiol.*.

[7] Steward C. D., Wallace D., Hubert S. K. (2000). Ability of laboratories to detect emerging antimicrobial resistance in nosocomial pathogens: a survey of Project ICARE laboratories. *Diagnostic Microbiology and Infectious Disease*.

[8] Lamichhane A., Sapkota S., Khadka S. (2020). Incidence of ESBL-producing Gram negative bacteria of lower respiratory tract infection in Bharatpur Hospital, Nepal. *Anti-Infective Agents*.

[9] Regmi R. S., Khadka S., Sapkota S. (2020). Bacterial etiology of sputum from tuberculosis suspected patients and antibiogram of the isolates. *BMC Research Notes*.

[10] Adhikari S., Khadka S., Sapkota S., Adhikaree N., Shrestha B., Parajuli A. (2020). Surgical site infections are the pool of antibiotic resistant bacteria: evidence from a tertiary hospital in Nepal. *Anti-Infective Agents*.

[11] Adhikari S., Khadka S., Sapkota S. (2021). Multi-drug resistant and extended spectrum *β*-lactamase producing Salmonella species isolated from fresh chicken liver samples. *Kathmandu University Medical Journal*.

[12] Sapkota S., Khadka S., Adhikari S., Parajuli A., Kandel H., Regmi R. S. (2020). Microbial diversity and antibiotic susceptibility pattern of bacteria associated with motorcycle helmets. *Int. J. Microbiol.*.

[13] Sapkota S., Adhikari S., Pandey A. (2019). Multi-drug resistant extended-spectrum beta-lactamase producing E. coli and Salmonella on raw vegetable salads served at hotels and restaurants in Bharatpur, Nepal. *BMC Research Notes*.

[14] Brolund A. (2014). Overview of ESBL-producing Enterobacteriaceae from a Nordic perspective. *Infection Ecology & Epidemiology*.

[15] Shakya P., Shrestha D., Maharjan E., Sharma V. K., Paudyal R. (2017). ESBL production among E. coli and Klebsiella spp. causing urinary tract infection: a hospital based study. *Open Microbiol. J.*.

[16] Bush K., Jacoby G. A. (2010). Updated functional classification of *β*-lactamases. *Antimicrobial Agents and Chemotherapy*.

[17] Jacoby G. A., Munoz-Price L. S. (2005). The new *β*-lactamases. *The New England Journal of Medicine*.

[18] Woerther P.-L., Burdet C., Chachaty E., Andremont A. (2013). Trends in human fecal carriage of extended-spectrum-lactamases in the community: toward the globalization of CTX-M. *Clinical Microbiology Reviews*.

[19] Bauernfeind A., Schweighart S., Grimm H. (1990). A new plasmidic cefotaximase in a clinical isolate of Escherichia coli. *Infection*.

[20] Laxminarayan R., Duse A., Wattal C. (2013). Antibiotic resistance--the need for global solutions. *The Lancet Infectious Diseases*.

[21] D’Andrea M. M., Arena F., Pallecchi L., Rossolini G. M. (2013). CTX-M-type *β*-lactamases: a successful story of antibiotic resistance. *International Journal of Medical Microbiology*.

[22] Adhikari S., Khadka S., Sapkota S. (2019). Prevalence and antibiograms of uropathogens from the suspected cases of urinary tract infections in Bharatpur Hospital, Nepal. *Journal of College of Medical Sciences-Nepal*.

[23] Duwadi K., Khadka S., Adhikari S., Sapkota S., Shrestha P. (2020). Bacterial etiology of wound exudates in tertiary care cancer patients and antibiogram of the isolates. *Infectious Diseases: Research and Treatment*.

[24] Forbes B., Sahm D., Weissfeld A. (2007). *Bailey & Scott’s diagnostic microbiology*.

[25] Clinical and Laboratory Standards Institute (CLSI) (2015). *Performance standards for antimicrobial susceptibility testing. twenty-fifth informational supplement*.

[26] Sambrook J., Russell D. (2001). *Molecular Cloning: A Laboratory Manual*.

[27] Stürenburg E., Kühn A., Mack D., Laufs R. (2004). A novel extended-spectrum *β*-lactamase CTX-M-23 with a P167T substitution in the active-site omega loop associated with ceftazidime resistance. *The Journal of Antimicrobial Chemotherapy*.

[28] Nepal K., Pant N. D., Neupane B. (2017). Extended spectrum beta-lactamase and metallo beta-lactamase production among Escherichia coli and Klebsiella pneumoniae isolated from different clinical samples in a tertiary care hospital in Kathmandu, Nepal. *Annals of Clinical Microbiology and Antimicrobials*.

[29] Abrar S., Ain N. U., Liaqat H., Hussain S., Rasheed F., Riaz S. (2019). Distribution of blaCTX−M, blaTEM, blaSHV and blaOXA genes in extended-spectrum-*β*-lactamase-producing clinical isolates: a three-year multi-center study from Lahore, Pakistan. *Antimicrobial Resistance and Infection Control*.

[30] Parajuli N. P., Maharjan P., Joshi G., Khanal P. R. (2016). Emerging perils of extended spectrum *β* -lactamase producing Enterobacteriaceae clinical isolates in a teaching hospital of Nepal. *BioMed Research International*.

[31] Shashwati N., Kiran T., Dhanvijay A. (2014). Study of extended spectrum *β*-lactamase producing Enterobacteriaceae and antibiotic coresistance in a tertiary care teaching hospital. *J. Nat. Sci. Biol. Med*.

[32] Doi Y., Wachino J., Arakawa Y. (2016). Aminoglycoside resistance: the emergence of acquired 16S ribosomal RNA methyltransferases. *Infectious Disease Clinics of North America*.

[33] Pokhrel R. H., Thapa B., Kafle R., Shah P. K., Tribuddharat C. (2014). Co-existence of beta-lactamases in clinical isolates of Escherichia coli from Kathmandu, Nepal. *BMC Research Notes*.

[34] Dahal R. H., Chaudhary D. K. (2018). Microbial infections and antimicrobial resistance in Nepal: current trends and recommendations. *Open Microbiol. J.*.

[35] Bush K. (2008). Extended-spectrum *β*-lactamases in North America, 1987-2006. *Clinical Microbiology and Infection*.

[36] Ozeki S., Deguchi T., Yasuda M. (1997). Development of a rapid assay for detecting gyrA mutations in Escherichia coli and determination of incidence of gyrA mutations in clinical strains isolated from patients with complicated urinary tract infections. *Journal of Clinical Microbiology*.

[37] Zeynudin A., Pritsch M., Schubert S. (2018). Prevalence and antibiotic susceptibility pattern of CTX-M type extended-spectrum *β*-lactamases among clinical isolates of gram-negative bacilli in Jimma, Ethiopia. *BMC Infectious Diseases*.

[38] Teklu D. S., Negeri A. A., Legese M. H., Bedada T. L., Woldemariam H. K., Tullu K. D. (2019). Extended-spectrum beta-lactamase production and multi-drug resistance among Enterobacteriaceae isolated in Addis Ababa, Ethiopia. *Antimicrobial Resistance and Infection Control*.

[39] Kizilca O., Siraneci R., Yilmaz A. (2012). Risk factors for community-acquired urinary tract infection caused by ESBL-producing bacteria in children. *Pediatrics International*.

[40] Raut S., Gokhale S., Adhikari B. (2015). Prevalence of extended spectrum beta-lactamases among Escherichia coli and Klebsiella spp isolates in Manipal Teaching Hospital, Pokhara, Nepal. *Journal of Microbiology and Infectious Diseases*.

[41] Ben-Ami R., Rodríguez-Baño J., Arslan H. (2009). A multinational survey of risk factors for infection with extended‐spectrum *β*‐lactamase–producing Enterobacteriaceae in nonhospitalized patients. *Clinical Infectious Diseases*.

